# Surgical Clipping Versus Endovascular Coiling in the Management of Intracranial Aneurysms

**DOI:** 10.7759/cureus.20478

**Published:** 2021-12-17

**Authors:** Rishab Belavadi, Sri Vallabh Reddy Gudigopuram, Ciri C Raguthu, Harini Gajjela, Iljena Kela, Chandra L Kakarala, Mohammad Hassan, Ibrahim Sange

**Affiliations:** 1 Surgery, Jawaharlal Institute of Postgraduate Medical Education and Research (JIPMER), Pondicherry, IND; 2 Research, Our Lady of Fatima University College of Medicine, Valenzuela, PHL; 3 Research, Tianjin Medical University, Tianjin, CHN; 4 Family Medicine, Jagiellonian University Medical College, Kraków, POL; 5 Internal Medicine, Jawaharlal Institute of Postgraduate Medical Education and Research (JIPMER), Pondicherry, IND; 6 Internal Medicine, Mohi-ud-Din Islamic Medical College, Mirpur, PAK; 7 Research, California Institute of Behavioral Neurosciences & Psychology, Fairfield, USA; 8 Research, K. J. Somaiya Medical College, Mumbai, IND

**Keywords:** endovascular coiling, surgical clipping, vascular surgery, neurosurgery, coiling, clipping, intracranial aneurysm

## Abstract

Intracranial aneurysms are pathological dilatations of intracranial arteries and prevail in around 3.2% of the general population. The worst outcome of an aneurysm is its rupture. Its prevention and management can be accomplished by two broad modalities: surgical clipping and endovascular coiling. This review has explored each of these approaches individually and has then directly compared them to provide a good understanding of their respective advantages and disadvantages over one another. Clipping is associated with a higher rate of occlusion of the aneurysm and lower rates of residual and recurrent aneurysms, whereas coiling is associated with lower morbidity and mortality and a better postoperative course. The risks and benefits of each of these procedures must be thoroughly examined in each case. This article has stressed the need to consider all contributing patient, procedure-related, surgeon-related, and hospital factors before arriving at a final decision to manage a specific case.

## Introduction and background

Intracranial or brain aneurysms are pathological dilatations of intracranial arteries that often occur at a branching point of the arteries [1]. The four broad types of aneurysms are saccular, fusiform, dissecting, and mycotic, of which saccular (or berry) aneurysms are the most common, accounting for around 90% [2]. The prevalence of intracranial aneurysms in the general population is approximately 3.2%, though most are asymptomatic [3-5]. Berry aneurysms are most commonly located in the anterior circulation, at the junction between the anterior communicating artery (ACOM) and the anterior cerebral artery (ACA), the bifurcation of the middle cerebral artery (MCA), and the junction between the posterior communicating artery (PCOM) and the internal carotid artery (ICA) [6-8]. A total of 15% occur in the posterior circulation, commonly at the basilar apex, at the junction between the basilar artery and the superior cerebellar (SCA) or anterior inferior cerebellar artery (AICA), and the junction of the vertebral and posterior inferior cerebellar arteries (PICA) [6-8]. The occurrence of aneurysms can be sporadic or familial [9]. Modifiable risk factors for acquired intracranial aneurysms include hypertension, chronic smoking, alcohol intake, and drug abuse (like cocaine and amphetamines) [10]. Familial conditions that have been associated with aneurysms include autosomal dominant polycystic kidney disease, Ehlers-Danlos syndrome type IV, Marfan syndrome, fibromuscular dysplasia, etc. [7]. The formation of an aneurysm is believed to be due to the interplay between hemodynamic stress and vascular insults, such as arteriosclerosis and atherosclerosis, with genetic predisposition playing an important role as well [11]. Although the majority of intracranial aneurysms are asymptomatic, among those that are symptomatic, the most common presentation is a severe headache, typically described as “the worst headache of my life” as a result of rupture and subarachnoid hemorrhage [12]. According to a meta-analysis published in 2005, the annual incidence of rupture ranges from 0.5% to 1.8% [13]. Rupture of an aneurysm is usually initiated by a trigger factor, which may be due to Valsalva maneuvers (like during defecation, coughing, or vomiting), sexual intercourse, strenuous physical exercise, or caffeine consumption. Other features of subarachnoid hemorrhage include nausea and vomiting, focal neurological deficits, loss of consciousness, and meningismus [14]. Those with unruptured aneurysms may present with focal neurological deficits, seizures, eye signs, etc., which occur depending on the aneurysm’s size and location [14]. Intracranial aneurysms are usually localized using computed tomography (CT) scan, magnetic resonance imaging (MRI), or digital subtraction angiography [15]. The importance of an interventional procedure lies in the prevention as well as the treatment of aneurysmal rupture and subarachnoid hemorrhage, which has a mortality rate of over 30%, while only 30% can return to an independent life [15]. Intervention revolves around two major modalities: surgical aneurysmal repair or "clipping" and endovascular aneurysmal repair or "coiling," with the possible addition of other devices such as stents [16]. Clipping is an invasive procedure in which a craniotomy is performed, and a clip is placed across the neck of the aneurysm to prevent inflow of blood into the dilated vessel, while coiling, being a minimally invasive procedure, involves passing a catheter via a peripheral artery into the cerebral circulation, and placing a coil precisely into the aneurysm, which induces clotting and blocks it off [17]. Surgeons often present variable opinions and disagreements when confronted with a choice between the two approaches. This review article aims to (i) explore the two surgical approaches individually regarding the patient profile, methods, advantages, and disadvantages, and (ii) compare and contrast these two approaches from a clinical perspective.

## Review

Surgical clipping

Surgical clipping as a treatment modality for intracranial aneurysms has been available for many decades, with the first case recorded in 1937 [18]. In surgical (or microsurgical) clipping, an open craniotomy is performed, the aneurysm dissected, and a clip placed to separate the aneurysm from the parent artery [17]. The procedure has evolved dramatically since its introduction. Up till the 1960s, it was performed under the naked eye and involved more considerable exposure of the brain with significant morbidity, thus leading McKissock et al. to the conclusion that conservative methods were a better treatment option than surgery [19]. Once microscopy was introduced to the field of neurosurgery in the late 1960s, clipping gave rise to a much better outcome with a drastic reduction in morbidity and mortality [20]. Gradually, other approaches were developed as replacements for the initial frontolateral approach that was used. These include the pterional, lateral suboccipital (LSO), lateral subtemporal, and orbitozygomatic approaches, which improved access to aneurysms located deep in the brain by reducing the distance between the incision site and the lesion [21-24]. It was not only the approach but also the clip in itself that underwent a series of changes. The clip used in the first recorded case in 1937 was a V-shaped clip made of silver, which, once applied, could not be removed, thus requiring extreme care and precision [25]. Modern-day clips are produced in various lengths, have varying angles and wider blade openings, are slimmer, have fenestrations or serrations to prevent slippage, and are made of non-corrosive alloys that are better tolerated than silver [26].

Various factors need to be taken into consideration before deciding on selecting surgical clipping for the management of intracranial aneurysms. They include the age of the patient, aneurysm size, and location [27]. A young patient with a small aneurysm located in the anterior circulation is the ideal candidate for clipping [27]. Posterior circulation aneurysms are challenging to access transcranially, and thus open surgery is not preferred. Better surgical outcomes were seen in younger patients (less than 50 years) with smaller aneurysms (less than 5 mm) located in the anterior circulation (treatment risk of 1%), as compared to treatment risks of 5% and 15% in anterior and posterior circulation aneurysms, respectively, among older patients (more than or equal to 70 years) with larger aneurysms (more than 10 mm) [28].

Quantifying its effectiveness can be done in terms of the occlusion rate and the morbidity and mortality rates. As per a paper published by David et al. in 1999, occlusion rates were around 90% [29]. A more recently published retrospective study involving 92 patients with MCA aneurysms, of which 54 underwent clipping, found the post-clipping occlusion rate to be 96.3% [30]. A meta-analysis published by Toccaceli et al. concluded that the long-term complete occlusion rate after surgery is 95%, thus strengthening the previous studies’ findings [31]. Since the advent of the less invasive method of coiling, clipping has become less preferred and is used more frequently for more giant aneurysms with more complex anatomy [32]. This has led to an effective reduction in the overall occlusion rate, which was 86.7% by CT imaging according to Van Lanen et al. in a study published in 2020 [32].

Operating time is an important parameter when it comes to any surgery as it almost always plays a role in the postoperative outcomes of the patient. Cha et al. conducted a retrospective review in which 122 patients with 137 aneurysms were analyzed between 2009 and 2011 [33]. The mean operating time by the classic pterional approach was 164.3 minutes, compared to the newer LSO approach, which on average lasted 117.1 minutes [33]. The same study found the mean duration of hospitalization to be nine days in the pterional group and 7.9 days for the LSO group, showing that the LSO approach is an excellent alternative to the more commonly used pterional approach. Another study compared operating times for clipping of paraclinoid aneurysms between the frontolateral and pterional approaches and found the frontolateral procedure to take 204.3 minutes, while the mean operating time of the pterional approach was 264.1 minutes [34]. We can see that the duration of the procedure varies widely based on the complexity of the location and the approach taken.

Clipping is an invasive procedure; it requires the skull to be opened and the aneurysm completely visualized. Thus, the rate of intraoperative difficulties and complications, such as inadequate exposure, injury to the brain matter, vessel injury leading to hemorrhage, and vessel occlusion causing ischemia, is relatively high. Taha et al. performed a retrospective analysis of all the cases that underwent surgical clipping at a single center between 2001 and 2004 and found the rate of periprocedural technical complications to be a significant 19.35% [35].

One significant advantage of clipping is the low rates of residual and recurrent aneurysms. Akyuz et al. analyzed 136 patients with 166 aneurysms and performed late post-operative follow-up at a mean of 46.6 months from the day of surgery; seven aneurysms had known residua, and no recurrence was seen [36]. Another more extensive, more recent retrospective study conducted by Brown et al. on 431 ruptured and 327 unruptured aneurysms showed 59 (7.8%) to have residual aneurysms on early post-operative imaging after one month, and only one recurrence was identified on post-operative imaging at a mean of 7.2 years post-discharge [37].

Various studies have been conducted to evaluate the survival, morbidity, and mortality rates of surgical clipping in the treatment of unruptured aneurysms. A retrospective study conducted by Britz et al. showed higher survival rates among those patients who underwent clipping and a 2.3% risk of death due to neurological causes (Table 1) [38]. The International Study of Unruptured Intracranial Aneurysms (ISUIA), headed by Wiebers et al. involving two groups of subjects hailing from the USA, Canada, and Europe, concluded that the overall morbidity and mortality ranged from 11% to 13.7% and 10.1% to 12.6% at 30 days and one year from the day of the surgery, respectively (Table 1) [39]. Ogilvy et al., in a study based out of Massachusetts General Hospital, found the overall mortality rate to be 0.8% and the morbidity rate to be 15.9% (Table 1) [28]. Morbidity in these studies included long-term neurological deficits, reformation of or residual aneurysms, bleeding, and ischemic stroke due to vessel occlusion.

**Table 1:** Results of extensive studies conducted on surgical clipping for unruptured and ruptured intracranial aneurysms.

Study	Type of study	Population	Aneurysm status	Sample size	Conclusion
Britz et al. (2004) [38]	Retrospective study	Washington, USA	Unruptured	4,619	Higher survival rates among those who underwent clipping, with a hazard ratio of 1.3. Those who survived beyond 30 days post-operative had a 2.3% chance of death due to neurologically related causes.
Wiebers et al. (2003) [39] - International Study of Unruptured Intracranial Aneurysms	Prospective study	USA, Canada, Europe	Unruptured	1,917	0.3-1.8% and 0.6-2.7% surgery-related deaths at 30 days and one-year post-operative, respectively. Overall morbidity and mortality were 11-13.7% and 10.1-12.6% at 30 days and one-year post-operative, respectively.
Ogilvy et al. (2003) [28]	Prospective study	Massachusetts, USA	Unruptured	493	Surgery showed overall morbidity and mortality of 15.9% and 0.8%, respectively. Small aneurysms in younger patients in the anterior circulation are associated with better outcomes.
Molyneux et al. (2002) [40] - International Subarachnoid Aneurysm Trial	Randomized controlled trial	Multiple centers internationally	Ruptured	2,143	Overall morbidity and mortality rate of 30.6% in the surgical group, and a 6.9% absolute risk reduction of dependency or death.

The mortality and morbidity rates are substantially higher when an aneurysm has ruptured before treatment by clipping. The International Subarachnoid Aneurysm Trial, a randomized controlled trial published by Molyneux et al. in 2002, found the overall morbidity and mortality rate to be 30.6% among those in the surgical treatment group, and the absolute risk reduction of dependency or death to be 6.9% (Table 1) [40]. The articles on surgical clipping are summarized in Table 1.

Institutional case volume also appears to play a role in determining the efficacy of the procedure. Rinaldo et al. reported a negative correlation between case volume and the overall complication rate [41]. Barker et al. conducted a retrospective cohort study on all United States (US) hospitals between 1996 and 2001 and saw that high-volume hospitals treating 20 or more cases per year discharge 84.4% of patients to their homes and are associated with a mortality rate of 1.6%, in comparison to 76.2% discharged to their homes and a mortality rate of 2.2% in low-volume hospitals (less than four cases per year) [42].

Methods have been developed over the years to improve outcomes after clipping. These include intraoperative ultrasonic flowmetry and Doppler sonography to assess vessel patency and intraoperative physiological brain monitoring to assess ischemic events during the procedure [43-46]. Thompson et al. recommend the consideration of certain specialized techniques, such as avoiding the use of fixed brain retractors and exposing the aneurysm through smaller, less invasive openings (like the “key-hole” approach) [40,47-49].

With the advent of the less invasive coil embolization, surgical clipping has taken a back seat in the management of intracranial aneurysms. Although new transcranial approaches and methods of clipping continue to be developed, coiling has become the preferred treatment of choice in many institutions. Within a few years of its first recorded use, endovascular treatment of aneurysms increased from 11% to 43% from 1998 to 2003 [50]. Despite this, clipping is still a vital modality when coiling is not possible, as in cases with very large aneurysms. Further development of the procedure is required to reduce morbidity and mortality rates and make it more patient-friendly.

Endovascular coiling

Coiling is a relatively recent treatment method for the management of intracranial aneurysms. The US Food and Drug Administration (FDA) approved the use of the Guglielmi detachable coil for the management of unruptured intracranial aneurysms in 1995 after Eskridge et al. analyzed 67 unruptured aneurysms that could not be surgically treated and found that select patients showed lower morbidity and mortality compared with conservative management [51]. Coiling involves passing a catheter via a peripheral artery, such as the femoral, into the cerebral circulation and precisely placing a coil into the aneurysm, which induces clotting, a process known as embolization [17].

The Raymond-Roy Occlusion Classification (RROC) has been the standard system for the evaluation of coiled aneurysms [52]. Here, class I is defined as complete occlusion, class II as residual neck, and class III as residual aneurysm. Mascitelli et al. proposed the Modified Raymond-Roy Classification (MRRC), dividing class III into class IIIa (contrast opacification within the coil interstices) and class IIIb (contrast opacification outside the coil interstices, along the residual aneurysm wall) [52]. They retrospectively found that class IIIa aneurysms were more likely to improve to class I or II than class IIIa aneurysms (83.3% vs. 14.9%) [52].

There has been a significant evolution of the technique since its inception. Nguyen et al., after having assessed all the aneurysms treated by coiling at a single center from 1992 to 2007, found the procedure-related rupture rate to be 11.7% for aneurysms less than or equal to 3 mm, as compared to just 2.3% in larger aneurysms [53]. The development of softer and smaller coils of around 1 mm in diameter has made it possible to treat smaller aneurysms without a high risk of rupture [54]. Similarly, coils with larger diameters have been developed to pack larger aneurysms using fewer coils. A single-center retrospective study compared the larger diameter Penumbra Coil 400 (PC400, Penumbra Inc., Alameda, CA) with the standard coil and found it to have a decreased procedure time (48 min vs. 64 min), fewer coils used (3.53 vs. 5.44), and increased packing density (31.7% vs. 24.8%) [55]. Another interesting evolvement is the bioactive coil as a possible replacement for the platinum coil. Its advantage lies in its coating, which has the ability to expand after contact with blood, thus potentially increasing packing density and reducing retreatment rates [54]. Despite this theoretical benefit, the Matrix and Platinum Science trial conducted by McDougall et al. did not find the Matrix coils (bioactive coils) superior to the bare metal coils, though their non-inferiority was demonstrated [56]. The Hydrogel Endovascular Aneurysm Treatment (HEAT) trial was a randomized controlled trial that compared the HydroCoil Embolic System (HES, MicroVention, Inc., Aliso Viejo, CA) (contains a platinum core with integrated hydrogel) with bare platinum coils (BPC), on both ruptured and unruptured 3-14 mm aneurysms [57]. A total of 4.4% in the HES group showed recurrence, as compared to 15.4% of the BPC group, showing the superiority of HES over BPC [57]. It was initially considered that large, complex, wide-necked, and fusiform aneurysms were untreatable by endovascular coiling [58]. With the advent of stent-assisted coiling, these aneurysms, especially wide-necked ones, have become amenable to endovascular treatment [59].

In the early years of coiling, comprehensive data on its value were unavailable because most studies were based on single centers with relatively smaller sample sizes. The Analysis of Treatment by Endovascular Approach of Nonruptured Aneurysms (ATENA) study conducted by Pierot et al. quashed most doubts related to the successful occlusion and morbidity and mortality rates associated with coiling (Table 2) [60]. The ATENA study was a prospective study published in 2008, including 649 patients with 1,100 unruptured aneurysms from multiple centers across France and Canada. They concluded that coiling has low morbidity and mortality rates and is a viable treatment option in the majority of patients. The relevant results of the ATENA study have been mentioned in Table 2.

**Table 2:** Results of the ATENA study, published in 2008. ATENA: Analysis of Treatment by Endovascular Approach of Nonruptured Aneurysms.

Parameter	Result
Success rate	The complete aneurysm occlusion rate was 59%.
Residual aneurysms	A neck remnant was found in 21.7% and an aneurysm remnant in 19.3%.
Failure rate	Failure was more in smaller aneurysms (1-6 mm) than larger ones (7-15 mm).
Complication rates	Procedure-related adverse events occurred in 15.4%, which included a 7.1% rate of thromboembolic complications. Periprocedural aneurysm rupture occurred in 2.6%. Neurological complications, primarily due to thromboembolic events, occurred in 5.4%. They were permanent in 2.6% and eventually led to death in 0.9%.
Morbidity and mortality	Morbidity and mortality rates after one month were 1.7% and 1.4%, respectively.

A major advantage of endovascular coiling is the accessibility to posterior circulation aneurysms. Of aneurysms, 8-15% are found in the posterior circulation, and their risk of rupture is higher than that of anterior circulation aneurysms [39,61-65]. Due to their deep location and the surrounding delicate structures like the brainstem, surgical approaches are relatively unsafe with a high complication rate [66]. Kim et al. retrospectively analyzed the outcomes of 621 posterior circulation aneurysms; 187 were treated by clipping and 434 by coiling [66]. Among patients with unruptured aneurysms, the rate of third nerve palsy was significantly lower in the endovascular group as compared to the surgical group (0.7% vs. 6.4%); rates of bleeding and ischemic events were not significantly different, but the rate of residual aneurysms was much lower in the surgical group (5.5% vs. 35.6% in the endovascular group). They also found that ruptured aneurysms in the SCA and PICA were more likely to be treated surgically, whereas those in the vertebral artery were more likely to be treated endovascularly. Similar trends in complication and residual rates were observed in ruptured aneurysms as those which were unruptured.

Many studies have been published to provide a further understanding of the effectiveness, safety, and morbidity related to coiling, but the one parameter that stands out in them all is the relatively low rate of complete occlusion. Gallas et al., in a retrospective study published in 2008 involving coiling of unruptured aneurysms, found the immediate total occlusion rate to be 70% and the subtotal occlusion rate to be 26.1%, the rest remaining non-occluded (Table 3) [67]. Morbidity was 14.4%, with ischemic complications occurring in 9% and perforation occurring in 2.6%. On long-term follow-up, 69.5% were completely occluded, 28.5% were subtotal, and 1.8% incompletely occluded. Occlusion rates were comparable in another retrospective study conducted by Bradac et al. in 2007, though it included both unruptured and ruptured aneurysms (Table 3) [68]. Complete occlusion was achieved in 64%, nearly complete in 34%, and incomplete in 2%. Periprocedural complications occurred in 13% and mainly were attributed to thromboembolisms and ruptures. The ruptured aneurysms group had morbidity and mortality of 1.1% and 2.2%, respectively, while those of the unruptured aneurysms group were 1.1% and 0%. In an older study published by Murayama et al. based on 916 aneurysms present in 818 patients, the complete occlusion rate was 55%, and a neck remnant was left behind in 35.4% (Table 3) [69]. Recanalization occurred in 20.9% and was related to the dome and neck size of the aneurysm. A total of 1.6% underwent delayed aneurysm rupture, and the majority were associated with large aneurysms. The ISUIA showed that the overall treatment-related morbidity and mortality were higher for patients with prior subarachnoid hemorrhage than for those without it (9.8% and 7.1%, respectively) (Table 3) [39]. Table 3 summarizes the conclusions of extensive studies based on coiling.

**Table 3:** Results of studies conducted to assess endovascular coiling of intracranial aneurysms and its occlusion, complication, and morbidity and mortality rates. ISUIA: International Study of Unruptured Intracranial Aneurysms; SAH: subarachnoid hemorrhage.

Study	Type of study	Population	Aneurysm status	Sample size	Conclusion
Gallas et al. (2008) [67]	Retrospective	Five institutions in France	Unruptured	302	Complete occlusion was seen in 70% and subtotal occlusion in 26.1%. Ischemic complications occurred in 9%. Treatment-related morbidity was 14.4%.
Bradac et al. (2007) [68]	Retrospective	A single center in Italy	Ruptured and unruptured	533	3.3% resulted in failure. Occlusion was complete in 64% and nearly complete in 34%. The complication rate was 13%, and the overall morbidity and mortality were 1.1% and 1.8%, respectively.
Murayama et al. (2003) [69]	Retrospective	A single center in California, USA	Ruptured and unruptured	818	Complete occlusion rate was 55%; a neck remnant was demonstrated in 35.4%. Overall morbidity and mortality rate was 9.4%, and overall recanalization rate was 20.9%.
Wiebers et al. (2003) [39] - ISUIA	Prospective	USA, Canada, Europe	Unruptured	451	After one year, the overall morbidity and mortality rate was 7.1% and 9.8% for patients without and with prior SAH, respectively.

Newer endovascular modalities have been developed that may be used in addition to or in place of coiling. Liquid embolics have been gaining the attention of late, especially the OnyxHD500 (eV3 Neurovascular, Irvine, CA), an ethylene-vinyl alcohol polymer [54]. Dalyai et al. tested this method on 21 patients with wide-neck aneurysms and achieved a 90% complete occlusion rate, thus deeming it to be an effective modality for treating wide-neck aneurysms or those that have failed treatment with detachable coils [70]. Another option is the use of stents to assist coiling. Higher occlusion rates and lower recurrence rates have been associated with stent-assisted coiling than coiling alone [71-73]. The downside to stent use, though, is the requirement of indefinite dual antiplatelet therapy, which comes with its own set of adverse effects [54]. Other developments are endoluminal flow diversion and aneurysmal neck reconstruction [74,75]. The Comaneci device (Rapid Medical, Yokneam, Israel), introduced to the US in 2019, is a retrievable stent that can be used along with coiling for better occlusion of wide-necked aneurysms without limiting blood flow [76]. Molina-Nuevo et al. concluded that the Comaneci device is a safe adjunctive treatment option for wide-necked aneurysms, with a complete occlusion rate of 88.8% and a major complication rate of 5.6% among 16 patients [77].

Thompson et al. recommend using endovascular coiling in the management of unruptured intracranial aneurysms in high-volume settings [27]. They also suggest that newer modalities such as endoluminal flow diversion and liquid embolics be considered only in carefully selected cases.

After discussing in-depth each of the primary treatment modalities individually, it is still unclear which has the edge over the other. Hundreds of studies have been conducted to establish a direct comparison between clipping and coiling; we look to delve deeper into some of these studies to understand further the advantages and disadvantages of one over the other.

Clipping versus coiling

Over the last 30 years, with the development of endovascular techniques, coiling has taken the front seat in the treatment of unruptured intracranial aneurysms; between 2001 and 2008, 34,054 aneurysms were treated by coiling while 29,866 were treated by clipping in the United States [78]. This exponential growth in the performance of endovascular procedures has led to several extensive studies being conducted over the years to compare them with the more historically established surgical methods.

One of the most critical comparative parameters is the occlusion rate. Schwartz et al., in a retrospective single-center study including 92 patients with MCA aneurysms, found the complete occlusion rate to be 96.3% after clipping and 78.9% after coiling [30]. The most considerable discrepancy in the rate of occlusion between clipping and coiling occurs in the treatment of large (15-25 mm) and giant aneurysms (>25 mm). A retrospective study published in 2020 by Choi et al. analyzing 112 patients with large and giant aneurysms found the complete occlusion rate after coiling to be just 36.3%, compared to 90.9% in the surgical group [79]. Thus, recurrence and retreatment rates were also significantly high at 46.8% and 31.9%, respectively. After aneurysm rupture, clipping still shows higher occlusion rates, with a single-center study based out of the US concluding that the rate of incomplete occlusion was 21.7% and 7.6% in the coiling and clipping groups, respectively [80]. We thus see that occlusion rates are relatively lower with coiling, corresponding to higher rates of residual aneurysms and future rupture.

Morbidity, mortality, and complication rates vary depending on the status of the aneurysm (ruptured or unruptured). Kim et al. conducted an extensive retrospective study that included all patients that underwent clipping or coiling for unruptured intracranial aneurysms in South Korea between 2008 and 2014 (Table 4) [81]. They concluded that there was no significant difference in the mortality rates between the two groups. However, the probability of retreatment within the following seven years was 4.9% after coiling and 3.2% after clipping. Another retrospective analysis based out of New York, USA, similarly showed no significant difference in the in-patient mortality rates or rates of readmission within 30 days, but the rate of discharge to rehabilitation and duration of hospital stay were higher in the clipping cohort (Table 4) [82]. McDonald et al. landed with the same results - periprocedural morbidity is significantly higher with coiling, but mortality rates are similar (Table 4) [83]. Contrary to this, Gonda et al. analyzed the treatment of 2,509 unruptured aneurysms and concluded that the perioperative mortality rate of the clipping group (2.3%) was significantly higher than that of the coiling group (1.1%) (Table 4) [84]. Of the clipping group, 20.4% required additional hospitalization for aneurysm repair compared to just 8.7% of the coiling group. A randomized controlled trial published by Darsaut et al. included 66 patients in the clipping group and 70 in the coiling group (Table 4) [85]. The overall one-year morbidity and mortality rate was 4.2% and 3.6%, respectively. New neurological deficits (23.1% of the clipping group vs. 8.7% of the coiling group) and hospitalization beyond five days (45.2% of the clipping group vs. 8.7% of the coiling group) occurred more often in those who underwent clipping.

**Table 4:** Summary of studies comparing the morbidity, mortality, and complication rates between surgical clipping and endovascular coiling. GCS: Glasgow Coma Scale.

Study	Study type	Population	Aneurysm status	Number of patients who underwent clipping	Number of patients who underwent coiling	Conclusion
Kim et al. (2018) [81]	Retrospective	South Korea	Unruptured	11,777 (44.6%)	14,634 (55.4%)	All-cause mortality rates of the clipping and coiling groups were similar (3.6% vs. 3.8%). At seven years from treatment, the probability of retreatment was 4.9% after coiling and 3.2% after clipping.
Bekelis et al. (2015) [82]	Retrospective	New York, USA	Unruptured	1,453 (31.3%)	3,190 (68.7%)	There was no significant difference in the rate of in-patient mortality or 30-day readmission. The rate of discharge to rehabilitation and length of stay were higher for the clipping cohort.
McDonald et al. (2013) [83]	Retrospective	USA	Unruptured	1,388 (28.1%)	3,551 (71.9%)	Periprocedural morbidity risk is significantly higher with clipping compared to coiling; in-hospital mortality risk is similar.
Gonda et al. (2014) [84]	Retrospective	California, USA	Unruptured	1,565 (60.5%)	944 (39.5%)	The clipping group showed a higher perioperative mortality rate (2.3% vs. 1.1% in the coiling group). Of those who underwent coiling, 20.4% required additional hospitalization for aneurysm repair compared to 8.7% of the clipping group. Coiling showed a significant cost advantage.
Darsaut et al. (2017) [85]	Randomized controlled trial	Canada	Unruptured	66 (48.5%)	70 (51.5%)	The clipping and coiling groups showed overall one-year morbidity and mortality of 4.2% and 3.6%, respectively. Clipping was associated with higher rates of new neurological deficits and hospitalization beyond five days.
McDonald et al. (2014) [86]	Retrospective	USA	Ruptured	1,228 (23.9%)	4,001 (76.1%)	There was no significant difference in in-hospital mortality between the two cohorts. Unfavorable outcomes like ischemic complications, neurologic complications, and discharge to long-term care were more frequent in the clipping group.
Bekelis et al. (2015) [87]	Retrospective	USA	Ruptured	1,206 (37.6%)	2,004 (62.4%)	There was no significant difference in the one-year postoperative mortality, discharge to rehabilitation, or 30-day readmission rate between the two groups. Those who underwent clipping stayed for 2.7 days longer on average compared to those who underwent coiling.
Lindgren et al. (2019) [88]	Nonrandomized controlled trial	Europe, USA, Australia	Ruptured	3,510 (45.8%)	4,148 (54.2%)	The case fatality 14 days after clipping was 8.2% and was 6.4% after coiling. Neither technique was found to be superior based on poor functional outcomes after 90 days.
Ayling et al. (2015) [89]	Randomized controlled trial	USA, Canada	Ruptured	165 (42%)	228 (58%)	Postoperative GCS scores were comparatively lower in the clipping group than in the coiling group.

Results of analyses of ruptured intracranial aneurysms are more variable. McDonald et al. found the in-hospital mortality to be similar in the clipping and coiling cohorts in a retrospective study published in 2014 that included 5,229 ruptured aneurysms, but clipping was associated with a significantly higher morbidity rate (Table 4) [86]. However, unfavorable outcomes such as ischemic complications, neurologic complications, discharge to long-term care, and other procedural complications occurred more frequently in those who underwent clipping. Similarly, Bekelis et al., in another retrospective study, found no significant difference in the one-year postoperative mortality, rate of discharge to rehabilitation, or 30-day readmission rate between the two techniques (Table 4) [87]. In contrast, Lindgren et al. published the results of a nonrandomized controlled trial in 2019, which showed the case fatality rate to be 8.2% and 6.4%, 14 days after clipping and coiling, respectively, although neither method was found to be superior based on poor functional outcome 90 days after the procedure (Table 4) [88]. Ayling et al. conducted a randomized controlled trial (165 underwent clipping and 228 underwent coiling) and found that clipping led to a poorer neurological outcome; postoperative Glasgow Coma Scale (GCS) scores were significantly lower in the clipping group than in the coiling group (Table 4) [89]. They also found that, on average, those who underwent clipping stayed in the hospital 2.7 days longer than those who underwent coiling. These large studies comparing clipping and coiling have been summarized in Table 4.

**Figure 1:**
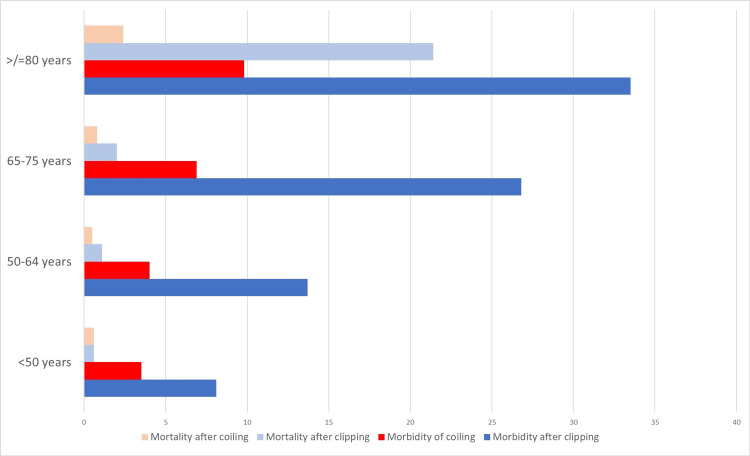
Bar graph comparing the age-wise morbidity and mortality rates between clipping and coiling. The bar graph is based on the results of Brinjikji et al. [78].

Brinjikji et al. measured the age-wise morbidity and mortality rates in a retrospective study analyzing all cases of unruptured intracranial aneurysms in the US between 2001 and 2008 [78]. Among patients younger than 50 years, there was no difference in the mortality rates, but clipping was associated with a significantly higher morbidity rate (8.1% vs. 3.5%). In all the other age groups (50-64 years, 65-79 years, and 80 years or older), both morbidity and mortality were found to be greater after clipping. The results have been summarized in Figure 1.

When choosing between clipping and coiling for each patient, the treating practitioner must also consider the duration of the hospital stay and the cost. In a retrospective study conducted by Higashida et al. on 2,535 unruptured intracranial aneurysm cases, the coiling procedure was associated with a shorter duration of hospitalization (4.5 vs. 7.4 days) and lower expenditure ($42,044 vs. $47,567) [90]. Similar findings were reported by Alshekhlee et al. - one day of hospitalization after coiling vs. four days after clipping and an average hospital bill of $38,116 for coiling and $42,070 for clipping [91].

As with many other conditions, the treating physician’s preferences also play an essential role. Fargen et al. released the results of a survey to understand the treatment preferences of practicing surgeons in the US in 2018 [92]. Among 211 physicians who filled out the survey, endovascular treatment was selected as the treatment of choice by 78% for ruptured aneurysms and 71% for unruptured aneurysms. Of the physicians, 80% use ISUIA data to understand the risks of complications and counsel the patient regarding the requirement of intervention. It was also found that clipping is likely to be the recommended option if the neurosurgeon is from an academic institution or has been practicing for a long period.

After having discussed these articles, we can see that among those that show significant differences between clipping and coiling, the majority conclude that lower morbidity and mortality and lower rates of discharge to long-term care are associated with coiling. However, the main factor against coiling is the relatively lower rates of complete occlusion related to it, which can be extrapolated to higher rates of residual and recurrent aneurysms.

Current recommendations are that both clipping and coiling are usable options for the treatment of both ruptured and unruptured intracranial aneurysms and both must be considered in each case [27]. The decision between the two is based on various factors - patient age, aneurysm size, location, and complexity, aneurysm status, the availability of resources at a particular center, and patient affordability and restrictions.

Limitations

This review takes into consideration these treatment options for both ruptured and unruptured aneurysms. However, we have not mentioned cases where a combination of clipping and coiling may be needed. Furthermore, an in-depth analysis of the new developments related to clipping and coiling has not been done due to insufficient data. We have also limited our review to the first presentation of these aneurysms, as management of recurrent or residual aneurysms requires the consideration of a vast number of patient, hospital, and procedure-related factors, which are beyond the scope of our discussion.

## Conclusions

Surgical clipping and endovascular coiling are the two standard treatment options for the treatment of intracranial aneurysms; however, the choice between the two is by no means an easy one and requires careful introspection for each unique case. The implication of this review is to provide a robust understanding of each of these treatment options, their efficacy, and the risk of complications, morbidity, and mortality, so that a treating practitioner may have more clarity in terms of decision-making. We spoke briefly about the evolution of each of these procedures, the patient demographics, and the rates of occlusion, complications, morbidity, and mortality. We cited various studies that have been performed to compare them directly. We have also discussed the newer developments in both the neurosurgical and endovascular fields in managing these aneurysms. The treatment choice must depend on patient factors (age, history of aneurysmal rupture, aneurysm status, presence of other comorbidities, and affordability), surgeon factors (expertise of the surgeon, years of experience, and their preference) as well as hospital factors (availability of resources like imaging, modern operating theatre, etc.). Although many comparative studies have been conducted, we recommend that more be performed with emphasis on stratifying the patient population based on these contributing factors. More data are also needed about the newer modalities that have recently been made available.
